# Subspace shrinkage in conjugate Bayesian vector autoregressions

**DOI:** 10.1002/jae.2966

**Published:** 2023-03-15

**Authors:** Florian Huber, Gary Koop

**Affiliations:** ^1^ University of Salzburg Salzburg Austria; ^2^ University of Strathclyde Glasgow UK

**Keywords:** Bayesian VAR, principal component regression, subspace shrinkage

## Abstract

Macroeconomists using large datasets often face the choice of working with either a large vector autoregression (VAR) or a factor model. In this paper, we develop a conjugate Bayesian VAR with a subspace shrinkage prior that combines the two. This prior shrinks towards the subspace which is defined by a factor model. Our approach allows for estimating the strength of the shrinkage and the number of factors. After establishing the theoretical properties of our prior, we show that it successfully detects the number of factors in simulations and that it leads to forecast improvements using US macroeconomic data.

## 1 INTRODUCTION

Macroeconomists are increasingly working with multivariate time series models involving large numbers of variables. Traditionally, factor models have been used (see, e.g., Geweke, 1977; Kaufmann & Schumacher, 2019; Stock & Watson, 2002 for the dynamic factor model [DFM] and Bernanke et al., 2005 for the factor‐augmented vector autoregression [FAVAR]). These typically use principal components (PCs) to extract the information in the large number of variables into a small number of factors thus avoiding overparameterization concerns. Starting with Banbura et al. (2010), many researchers have been simply including all the variables in a vector autoregression (VAR) and using Bayesian shrinkage priors to avoid overfitting (see, among many others, Carriero et al., 2019; Chan, 2020; Giannone et al., 2015, 2019; Hauzenberger et al., 2021; Huber & Feldkircher, 2019; Jarocinski & Mackowiak, 2017; Koop, 2013; Koop & Korobilis, 2019; Korobilis & Pettenuzzo, 2019).

How should the researcher decide whether to use a factor model or a large Bayesian VAR? This question can be answered through a comparison of their predictive performance in a pseudo out of sample forecasting exercise. Alternatively, marginal likelihoods can be used. But pseudo out‐of‐sample forecasting evaluation can be time consuming, and marginal likelihoods can be sensitive to the prior used. In this paper, we develop an alternative method for choosing between factor models and large VARs.

But why is there a need to choose between them when something in between might lead to better forecast performance? This is another question addressed in this paper. We propose a model which shrinks the VAR coefficients towards the implied coefficients of a PC regression model leading to a model which combines the two. We do so using a subspace shrinkage prior; see Shin et al. (2020). A conventional prior shrinks the posterior of a coefficient towards its prior mean, which is typically zero. In contrast, a subspace shrinkage prior is a prior on function spaces that shrinks towards a class of functions. In the present paper, we choose the class of functions to be PC regressions. 1 We stay in the class of conjugate priors (although we will discuss how other VAR priors can be accommodated), and thus, our methods are simple to implement. They do not require the use of computationally demanding Markov chain Monte Carlo (MCMC) methods, implying that these techniques are useful in very high dimensional models. We develop a method for estimating the weight put on the PC regression and the number of factors included in it. The result is a model that combines the large VAR with a factor model in an optimal way. Alternatively, output from our model can be used to select between the large VAR and the factor model and, if the latter is selected, determine the number of factors.

We consider two versions of our subspace VAR prior. First, the subspace prior can be combined with a conventional informative VAR prior such as the popular Minnesota prior (see Banbura et al., 2010; Doan et al., 1984; Kadiyala & Karlsson, 1997; Litterman, 1986; Sims & Zha, 1998 for a natural conjugate implementation). We demonstrate that results from such a model can be interpreted as a weighted average of the Minnesota prior VAR and the factor model. Second, the subspace prior can be combined with a noninformative VAR prior. The result is a new Bayesian VAR prior. In contrast to conventional priors which shrink towards plausible values for the VAR coefficients, our new prior shrinks towards the factor model.

Our approach is illustrated using synthetic and real data. In simulations, we consider two DGPs. The first one assumes that the data arises from a factor model. In this case, we find that our approach puts substantial weight on the subspace spanned by the PCs and accurately detects the number of factors if the true number of factors is small. This finding is independent of the model size. In larger dimensions, and for a larger number of true factors, our model slightly underestimates the true number of factors. The second DGP assumes that the data come from a VAR. In this case, the model puts very little weight on the PC‐based restrictions, leading to a standard VAR.

To further investigate the merits of our approach, we apply it to US macroeconomic data. In a forecasting exercise, the different priors that shrink the VAR towards a factor model improve upon a standard BVAR and the FAVAR. These improvements are pronounced during the global financial crisis and the Covid‐19 pandemic.

The main theoretical and empirical results in this paper involve a combination of a natural conjugate prior VAR with a single shrinkage parameter and a PC regression model. These choices are made for theoretical and computational simplicity. The Bayesian VAR literature considers many extensions of this homoskedastic natural conjugate case. In the latter part of this paper, we discuss how several of these extensions can be combined with subspace shrinkage. One extension that may be of particular empirical interest is the asymmetric conjugate VAR prior of Chan (2022). This allows for different VAR equations to have different shrinkage parameters. We show how this prior can be combined with subspace shrinkage and present empirical results which suggest the asymmetric conjugate prior combined with subspace shrinkage towards the factor model can lead to further improvements in forecast performance.

The remainder of the paper is structured as follows. Section 2 introduces the econometric framework. After providing a brief overview on conjugate Bayesian VARs and PC regressions in Section 2.1, we discuss our subspace shrinkage prior which can be used to force the coefficients of the VAR towards the restrictions implied by the PC regression in Sections 2.2 and 2.3. Section 2.4 discusses how our approach can be used to estimate the number of factors alongside the remaining model parameters. Section 3 provides simulation evidence that our model is able to detect the true number of factors while Section 4 applies our techniques to a big US macroeconomic dataset and illustrates its favorable forecasting properties. Section 5 discusses how alternative Bayesian VAR priors and extensions such as stochastic volatility can be incorporated in our techniques. This section also carries out an empirical exercise based on the asymmetric conjugate prior of Chan (2022). The final section summarizes and concludes the paper. Appendix S1 provides additional technical details, more simulation results, and precise information on the dataset used.

## 2 SUBSPACE SHRINKAGE IN VARs

### 2.1 Conjugate Bayesian VARs and factor models

Let ${\left\{Y_{t}\right\}}_{t=1}^{T}$ denote an M‐dimensional vector of macroeconomic and financial quantities. The number of time series can be large and, in addition, display substantial comovements. One popular approach of modeling this panel of time series is to assume $Y_{t}$ to follow a VAR( p) process:

(1) $$Y_{t}=A_{1}Y_{t-1}+\text{⋯}+A_{p}Y_{t-p}+\varepsilon_{t},$$

whereby $A_{j}\;(j=1,\ldots ,p)$ is an M×M‐dimensional coefficient matrix and $\varepsilon_{t}$ is a zero mean Gaussian shock with variance–covariance matrix Σ. 2 Equation (1) can be written as a multivariate regression model as follows:

$$Y_{t}=A^{\prime }X_{t}+\varepsilon_{t},$$

where $A={\left(A_{1},\ldots ,A_{p}\right)}^{\prime }$ and $X_{t}={\left(Y_{t-1}^{\prime },\ldots ,Y_{t-p}^{\prime }\right)}^{\prime }$ denote K(=Mp)×M and K×1 matrices, respectively. Stacking $Y_{t},X_{t}$ and $\varepsilon_{t}$ allows us to recast the model in full‐data form:

(2) Y=XA+ε,

with Y and ε are T×M with tth rows $Y_{t}^{\prime }$ and $\varepsilon_{t}^{\prime }$, respectively. X is T×K with tth row given by $X_{t}^{\prime }$.

Notice that the number of VAR coefficients in a=vec(A) is $k=pM^{2}$, which sharply increases with the number of endogenous variables and/or the number of lags. Because T is moderate for typical macroeconomic datasets, shrinkage is necessary to obtain well‐behaved estimates and to rule out implausible regions of the parameter space (e.g., regions that would imply explosive roots of the VAR process).

Bayesian priors on a are often used to provide such shrinkage. If M is large, natural conjugate priors are popular because they allow for fast computation. This arises because they preserve a convenient Kronecker structure for the posterior covariance matrix of a; see Chan (2020). The conjugate prior on a is specified conditionally on Σ and takes a Gaussian form:

(3) $$a|\Sigma ∼N\left(\text{vec}\left(\underline{A}\right),\Sigma ⊗\underline{V}\right).$$

Here, we let $\underline{A}$ denote a prior mean matrix of dimension K×M and $\underline{V}$ is a K×K matrix. The full conditional posterior distribution of a is also Gaussian with

$$\begin{array}{ll} a & |\Sigma ,Y∼N(\text{vec}(\bar{A}),\Sigma ⊗\bar{V}),\\ \bar{V} & ={\left(X^{\prime }X+{\underline{V}}^{-1}\right)}^{-1},\;\bar{A}=\bar{V}\left(X^{\prime }Y+{\underline{V}}^{-1}\underline{A}\right). \end{array}$$

The prior on Σ is inverted Wishart with prior degrees of freedom $\underline{\nu }$ and scaling matrix $\underline{S}$ which, when combined with the likelihood (and after integrating over a), yields a marginal posterior that also follows an inverted Wishart distribution whose posterior moments take a standard form (see, e.g., Chapter 21 of Chan et al., 2019).

A conventional Bayesian VAR prior such as the Minnesota prior would make particular choices for $\underline{A}$ and $\underline{V}$. An alternative would be to exploit the fact that the data might feature a factor structure. That is, the information in X might be characterized by a small number of q(q≪K) latent factors. These can be estimated using PCs which can be implemented through a singular value decomposition (SVD); see West (2003). The SVD writes $X=F_{q}L_{q}^{\prime }$ in terms of a T×q matrix $F_{q}$, which are the PCs, and a K×q matrix $L_{q}$, which is a matrix of factor loadings. If the matrix X is of rank q, this equation is exact. In general, if the rank of X exceeds q, $F_{q}L_{q}^{\prime }$ approximates X. Replacing X with $F_{q}L_{q}^{\prime }$ in Equation (2) shows that the corresponding matrix of regression coefficients $B=L_{q}^{\prime }A$ is of dimension q×M, a substantial reduction in the dimension of the state space. Using the Moore–Penrose inverse of $L_{q}$, $L_{q}^{†}$, allows us to express A in terms of B and the estimated loadings:

$$A=(L_{q}^{†})B.$$

This equation enables us to think about a factor model in terms of an otherwise unrestricted VAR with specific restrictions (which are driven by $L_{q}$) on the VAR coefficients. In a conventional factor model, these restrictions are always dogmatically imposed. In this paper, our goal is to introduce a shrinkage prior which softly pushes the elements in A towards the implied restrictions of the PC regression model.

### 2.2 Shrinking the flat prior VAR towards a factor model

Shrinking the regression model towards a subspace spanned by the PCs can be done in several ways. For instance, Oman (1982) shows how shrinkage estimators can be used to force an unrestricted regression model towards a projection on a subspace (such as the one spanned by the PCs) as opposed to the origin. This approach uses the eigenvalues of $X^{\prime }X$ to shrink coefficients towards the space spanned by the first q eigenvectors. Our approach is similar but relies on a modified variant of the functional Horseshoe prior stipulated in Shin et al. (2020). The basic version of the conjugate subspace shrinkage prior for VARs is given by

(4) $$a|\Sigma ∼N\left(0_{k},\Sigma ⊗{\left(\frac{\omega }{1-\omega }X^{\prime }(I_{T}-\Phi_{0})X\right)}^{-1}\right),$$

which has the same form of the general natural conjugate prior in (3) with $\text{vec}\left(\underline{A}\right)=0_{k}$ and $\underline{V}={\left(\frac{\omega }{1-\omega }X^{\prime }(I_{T}-\Phi_{0})X\right)}^{-1}$. Here, ω∈[0,1] is a shrinkage parameter, and the T×T matrix $\Phi_{0}=F_{q}{\left(F_{q}^{\prime }F_{q}\right)}^{-1}F_{q}^{\prime }$ is the projection of $F_{q}$. Recall that we obtain $F_{q}$ from the SVD of X. 3 We let ω be an unknown parameter and estimate it in a data‐based fashion as described below. The posterior is given in the preceding subsection with these particular choices of $\underline{A}$ and $\underline{V}$ inserted.

The prior in Equation (4) shrinks the estimates of the VAR coefficients towards the restrictions implied by the factor model. In Appendix S1.1, we show that the posterior mean of the regression function is a convex combination of the VAR fit, ΦY, and the fit of the PC regression, $\Phi_{0}Y$:

(5) $$𝔼(XA|Y,\omega )=\omega \Phi_{0}Y+(1-\omega )\Phi Y.$$

This result can be used to show that the resulting predictive distribution (or impulse responses) is weighted averages of the ones obtained from estimating an unrestricted VAR and a PC regression, both estimated using OLS. Larger values of ω imply estimates which are closer to the ones obtained from estimating a PC regression while values of ω closer to zero yield estimates closer to those of a noninformative prior Bayesian VAR.

Note that in the preceding material we are not incorporating any conventional Bayesian VAR prior such as the Minnesota prior. The prior given by Equation (4) is a new one which can be used if the researcher wishes to use a prior which only shrinks towards the factor model. We will use the acronym *subVAR‐Flat* to denote this prior which combines the subspace prior with a flat prior for the VAR coefficients. The fact that ω=0 yields a flat prior VAR illustrates an important aspect and potential shortcoming of this prior. Flat prior VARs tend to overfit unless M is very small and if K>T, as commonly occurs with large VARs, the OLS estimator will not be defined. Adding subspace shrinkage will ensure the posterior is proper, but small values of ω can potentially lead to overfitting. As we will document in our empirical results, using a noninformative prior for ω can lead to poor forecast performance in large VARs. Hence, the need for a suitable prior for ω. This will be provided below.

### 2.3 Shrinking the Minnesota prior VAR towards a factor model

Because the prior in Equation (3) is conjugate, we can easily add additional VAR priors to complement our subspace prior. In this subsection, we show how this can be done for the natural conjugate Minnesota prior as implemented in Banbura et al. (2010) or in Giannone et al. (2015).

The Minnesota prior has a long tradition as an empirically successful VAR prior; see Doan et al. (1984) and Litterman (1986). As shown, for example, in Sims and Zha (1998), an alternative way of obtaining the posterior arising from the Minnesota prior is to add a fictitious prior dataset, often referred to as dummy observations, of a particular form to the actual data. There is an equivalence between ordinary least squares results using this augmented dataset and posterior results using the Minnesota prior. This approach is used in, for example, Banbura et al. (2010) and in the present paper as described below. The key insight underlying our approach is that whereas in the preceding subsection Equation (5) established that the fitted regression line was a linear combination of the OLS and PC regression lines, in the present subsection, we establish it is a linear combination of OLS using the dummy‐augmented data with the PC regression line. For reasons discussed below, in this subsection, the relationship is approximate.

Let $\bar{Y}={\left(Y^{\prime },{\underline{Y}}^{\prime }\right)}^{\prime }$ and $\bar{X}={\left(X^{\prime },{\underline{X}}^{\prime }\right)}^{\prime }$ denote dummy‐augmented data matrices. The dummies $\underline{Y}$ and $\underline{X}$ can be specified to match features of the different priors in the Minnesota tradition. We assume that these dummies are parameterized by a hyperparameter ϑ, with values of ϑ close to zero implying strong shrinkage towards the prior mean. Our version of the Minnesota prior exactly follows Banbura et al. (2010), and more details are provided in Appendix S1.2.

We can add these dummies to Y and X and then combine it with our subspace shrinkage prior in (4). The posterior covariance matrix of the VAR coefficients then becomes

$$\bar{V}={\left({\bar{X}}^{\prime }\bar{X}+\underset{{\underline{V}}_{\Psi_{0}}^{-1}}{\underbrace{\frac{\omega }{1-\omega }{\bar{X}}^{\prime }\left[\left(\begin{array}{cc} I_{T} & 0\\ 0 & 0 \end{array}\right)-\left(\begin{array}{cc} \Psi_{0} & 0\\ 0 & 0 \end{array}\right)\right]\bar{X}}}\right)}^{-1}.$$

This equation implies that the prior variance depends on the sum of the prior covariance matrix implied by the Minnesota prior $\left({\underline{X}}^{\prime }\underline{X}\right)$, which is a function of ϑ, and the subspace shrinkage prior:

(6) $$\underline{V}={\left({\underline{X}}^{\prime }\underline{X}+{\underline{V}}_{\Psi_{0}}^{-1}\right)}^{-1}.$$

The resulting prior covariance matrix is a function of three hyperparameters. The overall tightness of the Minnesota prior ϑ, the number of factors q and the parameter that determines the weight on the PC regression ω. In Section 2.4, we discuss their treatment.

This completes our derivation of our second prior. This new VAR prior combines the Minnesota prior with shrinkage towards a PC regression model with projection matrix $\Phi_{0}$ which is calculated using the PCs of X. We label the resulting model *subVAR‐Minn*. It is worth stressing that it nests the subVAR‐Flat specification which is obtained by letting ϑ→∞.

Note that if we had calculated $\Phi_{0}$ using the dummy‐augmented dataset, then the relationship in (5) would hold, but using the dummy‐augmented dataset (i.e., replacing X and Y with $\bar{X}$ and $\bar{Y}$ in the formula). We do not do this, and therefore, the following is an approximate relationship:

(7) $$𝔼(XA|Y,\vartheta ,\omega )\approx \omega \Phi_{0}Y+(1-\omega )X{\left({\bar{X}}^{\prime }\bar{X}\right)}^{-1}{\bar{X}}^{\prime }\bar{Y}.$$

This result states that the posterior mean of the regression function is approximately a convex combination of the OLS fit of a PC regression and the posterior mean based on a Minnesota prior VAR. Intuitively speaking, if ϑ is set too tight, the Minnesota‐type prior overrules the subspace shrinkage prior.

We next establish that Equation (7) is a good approximation for reasonable values of the hyperparameters. 4 Our findings can be used to see how strong the Minnesota‐type shrinkage has to be before the relationship in (7) loses its usefulness as a guide to the theoretical properties of our prior. To this end, for different values of values of ϑ, we compute the average squared approximation error:

(8) $$\Xi (\theta ,\omega )=\frac{1}{M}\overset{M}{\underset{j=1}{\sum }}T^{-1}||X{\bar{A}}_{j}-\omega \Phi_{0}Y_{j}-(1-\omega )X{\left({\bar{X}}^{\prime }\bar{X}\right)}^{-1}{\bar{X}}^{\prime }{\bar{Y}}_{j}|{|}^{2},$$

with ${\bar{A}}_{j}$, $Y_{j}$, and ${\bar{Y}}_{j}$ denoting the jth column of the corresponding matrix and ||•|| denoting the Euclidean norm of a vector. This approximation error quickly approaches zero if ϑ becomes moderately large. If ω≈0, the approximation error also vanishes, since then, we obtain the Minnesota prior BVAR estimate. The interaction between ϑ and ω in determining Ξ(ϑ,ω) is highly nonlinear.

**FIGURE 1:**
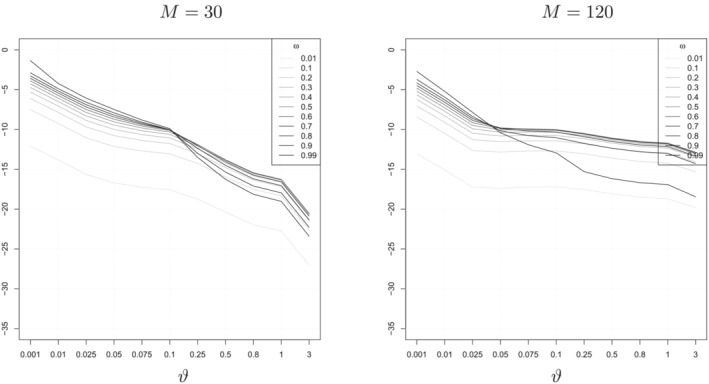
Log squared approximation error for M∈{30,120} and q=3.

These points are illustrated in Figure 1 which plots the (log) approximation error for different values of ϑ and ω using datasets simulated from different data‐generating processes (DGPs) for different values of M. The DGP is a DFM with q=3 and the factors evolving according to an AR(1) process with a full error variance–covariance matrix. 5

Figure 1 suggests that ω does not have a large effect on the approximation but that ϑ does. In particular, for values of ϑ>0.1, the log approximation error is less than −8 for all the different values of ω. In the next section, we will specify a prior on ϑ which allocates substantial mass to this region. It is worth stressing that even if ϑ is smaller than this, our prior is still a valid prior combining the Minnesota prior with the subspace prior, it is just that the posterior mean that results will deviate more from being a linear combination of a posterior mean using the Minnesota prior and a PC regression.

### 2.4 Selecting the number of factors and estimating the hyperparameters

Remember that the covariance matrix of the subVAR‐Minn prior given in Equation (6) depends on a choice for the number of factors (q), the weight attached to the VAR relative to the PC regression (ω) and the degree of shrinkage in the Minnesota prior (ϑ). 6 The posterior for these is

p(q,ω,ϑ|Y)∝p(Y|q,ω,ϑ)p(q,ω,ϑ).

Estimation is straightforward because the natural conjugate prior can be used to derive 7:

(9) $$p(Y|q,\omega ,\vartheta )\propto {\left(\frac{\left|\bar{V}\right|}{\left|\underline{V}\right|}\right)}^{\frac{M}{2}}\;|\bar{S}{|}^{-\frac{T+\underline{v}}{2}},$$

where $\bar{S}=\underline{S}+Y^{\prime }Y+{\underline{A}}^{\prime }{\underline{V}}^{-1}\underline{A}-{\bar{A}}^{\prime }{\bar{V}}^{-1}\bar{A}$ is the posterior scaling matrix of the inverse Wishart posterior of Σ. This can be multiplied by the prior to produce the posterior. For q, which is a discrete random variable, this leads to the discrete posterior. For the continuous random variables, ω and ϑ, we approximate their posteriors by evaluating them at a grid of discrete points. For all three of the parameters, we can then do Monte Carlo integration by sampling from the multinomial distributions that arise. Hence, our predictive densities reflect uncertainty in these parameters. This is similar to a strategy suggested in Giannone et al. (2015) for the Minnesota prior VAR but, as detailed below, avoids carrying out complex matrix operations during posterior simulation and thus offers substantial computational gains (at the cost of approximating a continuous posterior distribution using a discrete one).

It remains to specify the priors on the three hyperparameters. For the prior on the Minnesota shrinkage parameter ϑ, we follow suggestions in Giannone et al. (2015) and use a Gamma prior which we set to have mode 0.2 and standard deviation 0.4. This value implies that the approximation error in (8) is extremely small and the posterior mean of the model fit can be safely interpreted as a convex combination of the BVAR and the PC regression fit.

For the weight ω, we use a Beta prior: $\omega ∼B(c_{0},c_{1})$. In our empirical work, we consider two ways of specifying the hyperparameters $c_{0}$ and $c_{1}$. The first sets $c_{0}=c_{1}=1$, yielding a noninformative uniform prior on ω. The second prior sets $c_{0}=c_{0}\times M$ and $c_{1}=c_{1}\times M$, with $c_{0},c_{1}$ being scalars greater than zero. This choice implies that the prior mean on ω is equal to $c_{0}/(c0+c_{1})$ and the prior variance equals $\left(c_{0}\;c_{1})/({\left(c_{0}+c_{1}\right)}^{2}\;(M(c_{0}+c_{1})+1\right)$. In our empirical work, we set $c_{0}=8$ and $c_{1}=6$, yielding a prior mean on ω of around 0.6 and thus placing considerable mass on the factor model restrictions while the prior variance decreases in M. In large dimensions, this choice increasingly forces the model towards the factor restrictions but still provides sufficient flexibility for individual time series to exhibit VAR dynamics.

We assume a discrete uniform prior on the number of factors q:

$$q∼U(1,q_{0}),$$

which implies that all values up to $q_{0}$ (which denotes some integer smaller than K set by the researcher) are a priori equally likely. Other choices that utilize sample information (such as the eigenvalues of X) are in principle possible. It is worth noting that, in Bayesian factor analysis, selecting the number of latent factors is a difficult task. Common Bayesian solutions are based on reversible jump MCMC algorithms which treat the number of factors as an unknown quantity (see, e.g., Frühwirth‐Schnatter & Lopes, 2018; Lopes & West, 2004) or estimating overfitting factor models with a large number of factors and using Bayesian shrinkage priors on the loadings to estimate the effective number of factors; see Bhattacharya and Dunson (2011). However, the following section investigates, using simulated data, the simple and computationally efficient approach developed here. These simulations show that, even under a uniform prior, our approach selects the true number of factors successfully.

Two hyperparameters remain to be chosen. If we use a flat prior in combination with a subspace prior, we set $\underline{v}=M+2$ and $\underline{S}=\frac{1}{100}I_{M}$. If we use a Minnesota prior, we set $\underline{v}$ equal to the number of rows of $\underline{Y}$ and $\underline{S}={\left(\underline{Y}-\underline{X}\underline{A}\right)}^{\prime }\left(\underline{Y}-\underline{X}\underline{A}\right)$ (see Kadiyala & Karlsson, 1997).

It is also worth noting that, conditional on ω and ϑ, all quantities used in the Monte Carlo sampling of q can be precomputed and thus estimation of huge models (i.e., with M>100) is feasible. This requires specifying a grid for ω, θ, and q. In all our empirical work, we set the grid for $q\in \{1,\ldots ,\min(10,L^{*})\}$ with $L^{*}$ denoting the Ledermann bound. 8 The grid on ω is specified to go from 0.01 to 0.99 with a step size of 0.05. Finally, the grid on ϑ is {0.001,0.01,0.025,0.05,0.10,0.20,0.3,0.4,0.5,2,3,4,5}.

Equation (9) has the form of a marginal likelihood, conditional on the prior hyperparameters, and in our empirical work, we use this terminology, referring to our Monte Carlo method as marginal likelihood based. In high‐dimensional natural conjugate VARs, marginal likelihoods can be sensitive to the prior and occasionally difficult to estimate due to the need to take the determinant of the posterior covariance matrix. Accordingly, in our empirical work (which involves forecasting three variables of interest), we also investigate an alternative way of estimating q, ω, and ϑ. This is to use the Bayesian information criterion (BIC), which is an asymptotic approximation to the log of the marginal likelihood, for the three focus variables. To be precise, we take the three equations in the VAR for these variables which define a trivariate multivariate regression model (with unrestricted error covariance matrix). The BIC is based on the likelihood function for this model. It is evaluated at the posterior mean of the parameters. The penalty term in the BIC depends on the number of parameters which is the number of parameters in the trivariate regression model plus three (i.e., including ω, θ, and q.)

To summarize and introduce acronyms used in the empirical work, we have four models involving subspace priors (acronym subVAR). These involve two priors for the VAR coefficients: the noninformative one (flat) and the Minnesota prior (Minn). There are also two priors for ω: one noninformative (flat) and one informative. These are indicated by adding a 0 (flat) and 1 (tight) to the relevant labels of the VAR coefficient prior.

## 3 SIMULATED DATA EXERCISE

In this subsection, we investigate the properties of our model using synthetic data. We consider two DGPs. The first one assumes that the data arises from a factor model. The second DGP assumes that the data has been generated by a VAR with a single lag.

The first DGP is a DFM 9:

$$\begin{array}{ll} y_{t}=\Lambda f_{t}+\epsilon_{t},\;\epsilon_{t}∼N(0_{M},W), & \\ f_{t}=\Xi f_{t-1}+v_{t},\;v_{t}∼N(0_{q},\Omega ), & \end{array}$$

with Λ denoting an M×q matrix of factors, W is a diagonal matrix of measurement error variances, Ξ denotes a matrix of VAR coefficients, and Ω is a diagonal state‐innovation variance–covariance matrix with $\Omega =0.1^{2}\times I_{q}$. The initial state is set equal to $f_{0}=0_{q}$. In all our simulations, we assume that $\lambda_{ij}$, the (i,j)th element of Λ, is drawn from a Gaussian distribution with zero mean and variance 1 if i≠j. The upper q×q block is set equal to the identity matrix $I_{q}$. The autoregressive coefficients are simply given by $\Xi =0.95\times I_{q}$.

Instead of specifying Ω and W separately, we plug the state equation into the observation equation of the model and work out the implicit covariance matrix of the shocks to $y_{t}$, labeled Σ. This is done to ensure that the DGP is consistent with our VAR if ω=1. We then compute the lower Cholesky factor of $\Sigma =\Lambda \Omega \Lambda^{\prime }+W$, $A_{0}$, by simulating the off‐diagonal elements from a Gaussian distribution with zero mean and variance 0.01^2^ and the main diagonal elements are set equal to 0.01^2^.

The second DGP we consider is a VAR model:

$$y_{t}=Ay_{t-1}+\epsilon_{t},\;\epsilon_{t}∼N(0_{M},\Sigma ),$$

where $\Sigma =A_{0}A_{0}^{\prime }$ with the off‐diagonal elements of $A_{0}$, again, coming from a Gaussian distribution with zero mean and variance 0.01^2^ while the diagonal elements are again equal to 0.01. The off‐diagonal elements in A are obtained from independent Gaussians with variance 0.1^2^ and the diagonal elements are set equal to 0.8.

For both DGPs, we simulate T=500 observations from moderate ( M=30), large ( M=60), and huge ( M=120) datasets. For the first DGP, we vary the number of factors q∈{1,3,6,8}. All simulations are repeated 100 times, and in Tables 1 and 2, we report averages of posterior means/medians across these replications.

**TABLE 1:** Simulation results for different values of q and M: factor model DGP.

	subVAR‐Minn0				subVAR‐Minn1				subVAR‐Flat0				subVAR‐Flat1		
q=	1	3	6	8	1	3	6	8	1	3	6	8	1	3	6	8
Posterior mean of q															
M=30	1.00	3.00	6.00	8.00	1.00	3.00	5.96	8.00	1.00	3.00	6.00	7.96	1.00	3.00	6.00	8.00
M=60	1.00	3.00	5.92	7.72	1.00	2.96	5.80	7.48	1.00	3.00	5.88	7.92	1.00	3.00	5.88	7.32
M=120	1.00	2.04	3.20	3.68	1.00	1.84	2.72	3.16	1.00	1.92	3.08	3.64	1.00	1.72	2.76	3.36
Posterior mean of ω															
M=30	0.99	0.99	0.99	0.99	0.79	0.77	0.75	0.75	0.99	0.99	0.99	0.99	0.79	0.77	0.76	0.75
M=60	0.99	0.99	0.99	0.99	0.86	0.86	0.83	0.81	0.99	0.99	0.99	0.99	0.86	0.86	0.83	0.81
M=120	0.99	0.99	0.99	0.99	0.91	0.90	0.86	0.86	0.99	0.99	0.99	0.99	0.91	0.89	0.86	0.86

*Note*: subVAR denotes the VAR coupled with the subspace shrinkage prior, Minn is the combination between subspace and Minnesota shrinkage while flat is the subspace shrinkage prior without additional shrinkage. The 0 and 1 attached to the respective label indicate a flat (0) or informative (1) prior on ω. Each number is based on computing the mean of posterior medians across 100 replications from the respective DGPs.

**TABLE 2:** Simulation results for different values of q and M: VAR‐DGP.

	subVAR‐Minn0	subVAR‐Minn1	subVAR‐Flat0	subVAR‐Flat1
Posterior mean of q				
M=30	1.00	1.00	1.00	1.00
M=60	1.00	1.00	1.00	1.00
M=120	1.00	1.00	1.00	1.00
Posterior mean of ω				
M=30	0.10	0.14	0.10	0.14
M=60	0.16	0.21	0.16	0.21
M=120	0.31	0.35	0.31	0.35

*Note*: subVAR denotes the VAR coupled with the subspace shrinkage prior, Minn is the combination between subspace and Minnesota shrinkage while flat is the subspace shrinkage prior without additional shrinkage. The 0 and 1 attached to the respective label indicate a flat (0) or informative (1) prior on ω. Each number is based on computing the mean of posterior medians across 100 replications from the respective DGPs. For q, we use the posterior median as our point estimate while for ω we use the posterior mean.

It can be seen that all of the versions of our subVAR prior are doing a good job of estimating the correct number of factors. It is only in the least parsimonious cases (i.e., DGPs with M=120 and q=6 or 8) where it is considerably underestimating the number of factors. This is due to the large VAR providing some of the fit, leaving less for the PC regression to explain. Put differently, if the number of observations is small relative to the number of coefficients (which is the case for M=120), the information in the likelihood is not sufficiently informative to decide on whether a PC regression or a VAR fits the data better. We substantiate this claim in Appendix S2 which reruns the simulations but sets T=2500. In this case, the model is able to learn the true number of factors extremely well.

Nevertheless, it is worth stressing that our approach, in huge dimensions, strikes a balance between a model that includes many factors or a model that features few factors but rich deviations from the common factor structure using the VAR part. In the context of these very large models, slight overshrinkage is better than the overfitting which would have occurred if the prior had failed to shrink enough.

Another reason for overshrinkage in very large VARs could relate to our assumption of a single ω. In the factor literature, the importance of column‐specific shrinkage for identifying the true number of factors has been noted; see, for example, Legramanti et al. (2020). In Section 5.2, we consider an extension of the natural conjugate prior developed in Chan (2022) which allows for column‐wise shrinkage.

Tables 1 and 2 also provide evidence on the estimation of ω. When the true DGP is a factor model, the former table indicates that ω is often close to one (and always much greater than 0.5) whereas if the DGP is a VAR, the latter table finds that most weight is placed on the VAR (with ω being close to zero and always smaller than 0.5). In this case, the estimated values of ω increase somewhat with model dimension. When the informative prior on ω which favors the factor model is used, its posterior is slightly pulled towards the factor model but still allocates most of the weight to the VAR.

## 4 FORECASTING USING US MACROECONOMIC DATA

### 4.1 Data

We use a large set of 166 quarterly macroeconomic variables taken from the St. Louis Fed's FRED data base (fred.stlouisfed.org) and discussed in McCracken and Ng (2020). These are listed in Table 6 in Appendix S3. Variables are transformed to stationarity following recommendations there. Our forecasting results focus on three variables of interest: GDP growth (based on real GDP growth, GDPC1), the Fed Funds rate (FEDFUNDS), and inflation (based on the consumer price index, CPIAUCSL).

The data run from 1960:Q1 to 2020:Q3, and in our forecasting exercise, the evaluation period is from 1990:Q3 to 2020:Q3. We adopt a recursive forecasting design. We use the initial estimation period (1960:Q1 to 1990:Q2) to produce one‐ and iterated four‐quarter‐ahead forecast distributions for 1990:Q3 and 1991:Q2, respectively. After obtaining these, we expand the initial estimation period by one observation until we reach the end of the sample.

### 4.2 Models

In Section 2.4, we defined four models involving the subspace shrinkage prior (i.e., subVAR‐Minn0, subVAR‐Minn1, subVAR‐Flat0, and subVar‐Flat1). For each of these models, we present results for datasets of four different sizes: small (S, 12 variables), medium (M, 22 variables), large (L, 78 variables), and extra large (XL, 166 variables). Table 6 in Appendix S3 lists which variable belongs in which category.

For comparison, we also present results for Minnesota prior VARs (implemented by setting ω=0 in the subVAR‐Minn) and a FAVAR. The FAVAR is simply a VAR, using the same Minnesota prior as in our subspace shrinkage VAR, for the three variables of interest and a number of PCs extracted from the remaining time series. The number of PCs is chosen by retaining the PCs with standard deviations greater than unity. The shrinkage parameter ϑ is simulated in the same way as in our subspace shrinkage VAR. This implies that we use the marginal likelihood and the BIC to construct a discrete approximation to the conditional posterior of ϑ. In all models, we set the number of lags equal to p=2.

### 4.3 Summary of forecasting results

We begin by summarizing the results of our pseudo out of sample forecasting exercise in Table 3. This table contains root mean squared forecast errors (RMSFEs) and averages (over time) of log predictive likelihoods (LPLs) for our three variables of interest and for two different forecast horizons. The RMSFEs are ratios between the RMSFEs of a given model and the Minnesota VAR while the LPLs are differences in the LPL between a given model and the Minnesota VAR (both for a given model size).

**TABLE 3:** Forecasting results across focus variables, models, and forecast horizons.

		FEDFUNDS						CPIAUCSL						GDPC1					
		subVAR				FAVAR	BVAR	subVAR				FAVAR	BVAR	subVAR				FAVAR	BVAR
		Minn0	Minn1	Flat0	Flat1			Minn0	Minn1	Flat0	Flat1			Minn0	Minn1	Flat0	Flat1		
	Marginal likelihood to estimate q, ω, and ϑ																		
	One‐quarter‐ahead																		
	S	1.00	0.93	1.02	0.95	1.07	0.58	0.99	0.98	0.98	0.98	1.00	1.22	0.99	0.98	0.99	0.99	1.00	1.14
		(−0.01)	(0.03)	(0.02)	(0.09)	(−0.55)	(−0.87)	(0.06)	(0.11)	(0.09)	(0.14)	(0.36)	(−2.01)	(−0.05)	(−0.03)	(−0.02)	(0.05)	(−0.02)	(−1.62)
	M	1.02	0.91	1.11	1.04	1.17	0.65	0.99	0.97	1.02	0.99	0.98	1.26	1.00	0.99	1.03	1.01	1.03	1.14
		(0.02)	(0.07)	(−0.03)	(0)	(−0.63)	(−0.86)	(−0.03)	(0.12)	(0.05)	(0.20)	(0.71)	(−2.33)	(0.01)	(0.01)	(−0.03)	(−0.03)	(0.09)	(−1.65)
	L	0.97	0.62	1.87	0.96	1.3	0.74	0.98	0.86	1.33	0.97	0.99	1.32	0.98	0.93	1.38	1.02	1.01	1.2
		(0.13)	(0.51)	(−0.56)	(−0.08)	(−0.54)	(−1.24)	(0.53)	(2.27)	(1.61)	(2.44)	(2.38)	(−4.09)	(0.01)	(0.11)	(−0.42)	(−0.31)	(0.1)	(−1.73)
	XL	0.97	0.68	1.45	1.21	1.37	0.74	0.98	0.87	1.09	0.97	1.04	1.35	1.00	0.92	1.10	0.97	1.02	1.21
		(0.81)	(1.53)	(0.64)	(−0.01)	(0.41)	(−2.27)	(0.66)	(2.66)	(3.33)	(2.94)	(3.42)	(−5.2)	(0.08)	(0.53)	(0.01)	(−0.4)	(0.61)	(−2.27)
	One‐year‐ahead																		
	S	1.03	1.03	1.07	1.00	1.07	0.48	1.01	1.00	1.01	1.01	1.03	1.14	0.99	1.00	0.99	1.00	1.00	1.12
		(−0.06)	(0.07)	(−0.21)	(−0.09)	(−0.47)	(−1.07)	(0.02)	(−0.01)	(−0.05)	(−0.01)	(0.02)	(−1.6)	(0.01)	(0.01)	(−0.09)	(−0.06)	(−0.01)	(−1.63)
	M	1.03	0.96	1.19	1.09	1.01	0.53	1.00	1.00	1.01	1.00	1.05	1.15	1.01	0.99	1.00	1.00	1.01	1.12
		(−0.03)	(0.08)	(−0.45)	(−0.39)	(−0.64)	(−1.04)	(−0.02)	(−0.12)	(0.09)	(0.10)	(0.08)	(−1.75)	(−0.05)	(−0.02)	(−0.17)	(−0.16)	(−0.03)	(−1.6)
	L	0.92	0.66	7.51	7.06	1.28	0.69	0.99	0.95	3.58	5.04	1.07	1.18	0.99	0.98	6.29	7.11	1.08	1.13
		(0.04)	(0.32)	(−3.63)	(−5.51)	(−1.67)	(−1.56)	(0.02)	(0.05)	(−3.41)	(−5.36)	(−1.21)	(−1.57)	(0)	(−0.06)	(−3.7)	(−5.68)	(−0.93)	(−1.66)
	XL	0.84	0.54	>10	>10	1.24	0.86	0.99	0.95	>10	>10	1.08	1.19	0.97	0.98	>10	>10	1.04	1.13
		(−0.10)	(−0.88)	(−6.56)	(−10.54)	(−1.72)	(−1.45)	(−0.08)	(−0.72)	(−6.61)	(−10.29)	(−1.16)	(−1.54)	(−0.19)	(−1.06)	(−7.00)	(−10.78)	(−1.04)	(−1.51)
	BIC for the three focus variables used to estimate q, ω, and ϑ																		
	One‐quarter‐ahead																		
	S	0.96	0.91	0.99	0.9	0.95	0.66	0.99	0.96	0.99	0.96	0.98	1.24	0.99	0.99	1	0.99	0.99	1.16
		(0.04)	(0.07)	(0.01)	(0.08)	(−0.47)	(−0.96)	(0.09)	(0.22)	(0.07)	(0.2)	(0.45)	(−2.1)	(−0.04)	(−0.07)	(−0.13)	(−0.07)	(−0.07)	(−1.6)
	M	0.93	0.75	0.94	0.74	0.87	0.85	0.98	0.9	0.97	0.89	0.9	1.37	0.98	0.93	0.98	0.94	0.96	1.21
		(0.05)	(0.2)	(0.07)	(0.22)	(−0.45)	(−1.04)	(0.25)	(0.65)	(0.22)	(0.71)	(1.33)	(−2.94)	(−0.05)	(0.02)	(0.03)	(0.02)	(0.13)	(−1.68)
	L	0.94	0.83	1.81	1.18	1.38	0.71	0.98	0.93	1.3	1.06	0.99	1.30	0.98	0.95	1.35	1.10	1.01	1.19
		(0.2)	(0.32)	(−0.41)	(−0.21)	(−0.65)	(−1.13)	(0.36)	(0.8)	(1.31)	(1.74)	(1.79)	(−3.48)	(−0.06)	(0.16)	(−0.35)	(−0.28)	(0.07)	(−1.74)
	XL	0.95	0.83	1.42	1.18	1.41	0.72	0.97	0.91	1.08	0.98	1.08	1.34	0.98	0.93	1.11	1.02	1.04	1.20
		(0.59)	(0.81)	(−0.24)	(−0.89)	(−0.23)	(−1.63)	(0.19)	(1.98)	(2.78)	(2.41)	(3.05)	(−4.85)	(0.21)	(0.51)	(−0.21)	(−0.65)	(0.59)	(−2.26)
	One‐year‐ahead																		
	S	1.01	0.97	1.02	1	0.98	0.51	0.99	0.98	0.99	0.98	1.01	1.17	1.01	1.00	1.00	1.00	1.00	1.12
		(0.06)	(0.28)	(−0.01)	(0.26)	(−0.16)	(−1.39)	(0.02)	(0.04)	(0.01)	(0.08)	(0.09)	(−1.69)	(0)	(0.06)	(−0.03)	(0.05)	(0.06)	(−1.66)
	M	0.83	0.55	0.90	0.56	0.54	0.96	0.95	0.91	0.97	0.91	0.95	1.28	0.95	0.92	0.96	0.93	0.94	1.21
		(0.11)	(0.61)	(0.08)	(0.58)	(0.05)	(−1.72)	(0.04)	(0.02)	(0.02)	(−0.01)	(0.05)	(−1.74)	(0.07)	(0.20)	(0.04)	(0.17)	(0.22)	(−1.83)
	L	0.87	0.74	>10	>10	1.23	0.65	0.99	0.99	3.86	>10	1.06	1.16	0.99	0.98	6.47	>10	1.06	1.13
		(0.06)	(0.27)	(−4.17)	(−4.9)	(−1.74)	(−1.49)	(−0.03)	(−0.07)	(−3.94)	(−4.7)	(−1.26)	(−1.53)	(−0.05)	(−0.05)	(−4.25)	(−5.05)	(−0.93)	(−1.63)
	XL	0.85	0.66	>10	>10	1.34	0.77	0.98	0.96	>10	>10	1.1	1.18	0.99	0.99	>10	>10	1.04	1.12
		(−0.24)	(−0.87)	(−8.09)	(−10.1)	(−1.7)	(−1.45)	(−0.17)	(−0.66)	(−8.12)	(−9.87)	(−1.13)	(−1.57)	(−0.27)	(−1.00)	(−8.42)	(−10.28)	(−0.93)	(−1.62)

*Note*: subVAR denotes the VAR coupled with the subspace shrinkage prior, Minn is the combination between subspace and Minnesota shrinkage while flat is the subspace shrinkage prior without additional shrinkage. The 0 and 1 attached to the respective label indicate a flat (0) or informative (1) prior on ω. FAVAR is a VAR in the three focus variables augmented with principal components and BVAR refers to a Minnesota VAR. The numbers are relative root mean squared forecast errors (RMSFEs) between a given model and the Minnesota VAR while the numbers in parentheses are differences in average log predictive likelihoods (LPLs) to the Minnesota VAR. The numbers in the BVAR columns include the actual RMSFEs and LPLs of the Minnesota VAR.

Before discussing our subVAR models, consider the comparison between the Minnesota prior VAR and the FAVAR. For some variables, forecast horizons, and model sizes, the VAR yields more precise forecasts. That is, for the interest rate, it consistently forecasts better and for inflation and GDP growth for larger models at longer horizons, its forecasts tend to be better than the ones of the FAVAR. But for other cases, the factor model outperforms the Bayesian VAR. This result raises the possibility that an approach such as ours, which combines the two, could lead to better overall forecast performance than either the BVAR or FAVAR individually, and with several exceptions discussed below, this is what we find.

Consider first the most informative subVAR model which uses the Minnesota prior on the VAR coefficients and the informative prior on ω (Minn1). With some exceptions, this model is yielding forecasts which are better than the predictions produced by the BVAR and are often the best ones overall. The main exceptions are the 1‐year‐ahead GDP growth predictions which are marginally worse than the BVAR benchmark. However, this is a case where the FAVAR and some of the less informative subVAR approaches are forecasting substantially worse than the BVAR.

Consider now the second most informative subVAR approach (Minn0) which retains the Minnesota prior for the VAR coefficients but uses a noninformative prior on ω. Its forecasts are comparable with those of Minn1, but overall are slightly worse. But clearly, results are robust to the prior on ω. Both of these Minnesota prior subVAR approaches are forecasting well most of the time and even the few exceptions reveal only slight deterioration in forecast performance relative to the BVAR benchmark.

Using a noninformative prior for the VAR coefficients, however, goes wrong in some cases in larger models. In one sense, this is unsurprising. Noninformative priors work poorly in large VARs because they suffer from severe overparameterization problems and can lead to posteriors which allocate nonnegligible weight to the nonstationary region of the parameter space. In general, this is a key reason why prior shrinkage is used with VARs, particularly in large VARs and flat priors are often avoided. Adding subspace shrinkage will not necessarily help rule out nonstationary regions of the parameter space. It is dependent on the PCs which may be nonstationary. Hence, in some datasets, it may help, but in others not. In our dataset, the subspace prior shrinking towards the factor model is clearly not strong enough in the L and XL models to ensure all of the posterior lies in the stationary region. One might have hoped that estimates of ω would have been pulled towards 1 in these cases, leading to results similar to the FAVAR but (unless we use an extremely dogmatic prior on ω) this is not happening in the larger models. Similar to the results based on synthetic data, this is because the larger (unrestricted) VARs explain the majority of variation in the data and leave little variation to explain for the factor model, yielding posterior estimates of ω close to zero. However, the subVAR‐Flat models are performing well for our small‐ and medium‐sized models and for the one‐quarter‐ahead forecast horizon for the larger models. And it is only the iterated 1‐year‐ahead forecasts that are deteriorating. This suggests that this approach might be found useful by researchers working with VARs up to a dimension of approximately 20 who wish to avoid the use of standard BVAR priors such as the Minnesota prior, particularly if the focus is on short‐term forecasts.

### 4.4 A deeper examination of forecast performance of subspace VAR methods

To examine more deeply the properties of our subVAR prior, in this subsection, we provide plots over time of predictive Bayes factors against the Minnesota prior VAR. Moreover, we investigate how the estimates of the prior hyperparameters q, ω, and ϑ evolve over the hold‐out period. For the sake of brevity, we present results only for one‐quarter‐ahead forecasts.

**FIGURE 2:**
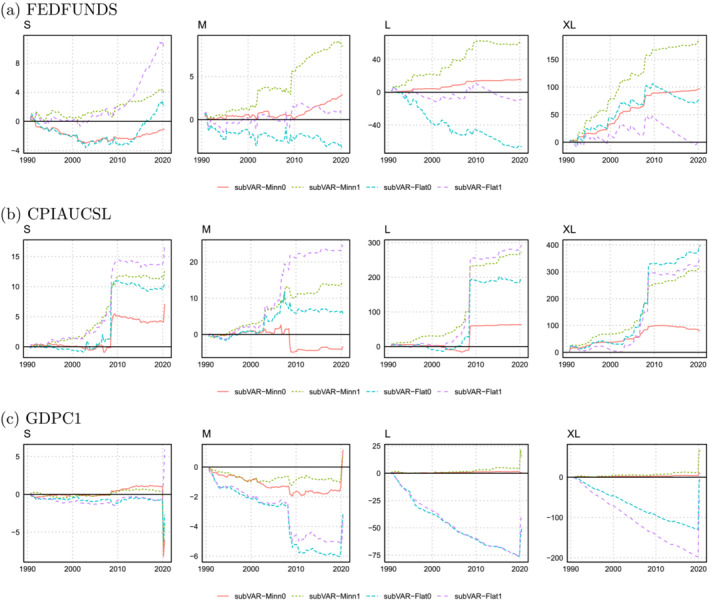
Evolution of the log predictive Bayes factor between subVAR and the Minnesota VAR across focus variables when the marginal likelihood is used to select q, ϑ, and ω.

**FIGURE 3:**
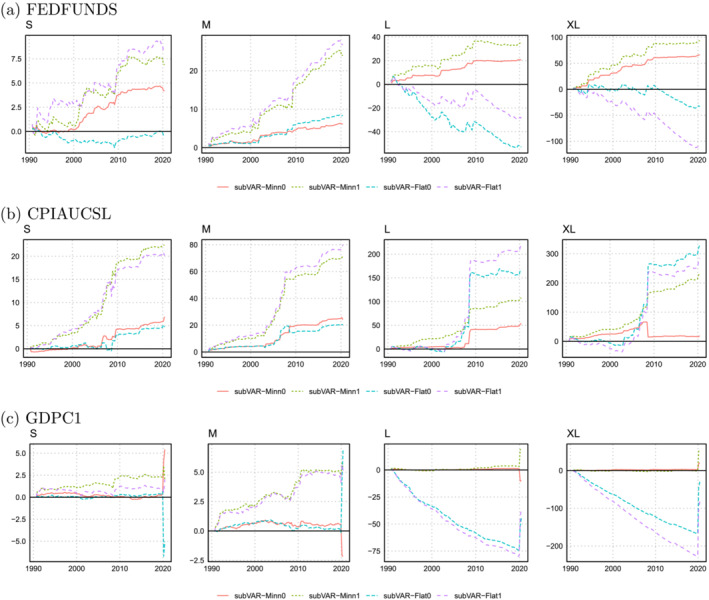
Evolution of the log predictive Bayes factor between subVAR and the Minnesota VAR across focus variables when the BIC over the three focus variables is used to select q, ϑ, and ω.

Figures 2 and 3 plot the log predictive Bayes factors for the three variables being forecast for the four subspace VAR priors. Figure 2 uses the marginal likelihood for all the variables in the model to estimate the prior hyperparameters, and Figure 3 uses the BIC for the three variables of interest. The overall best performance of the priors which combine subspace shrinkage with the Minnesota prior can be seen in both figures. An examination of the main exception to this pattern is informative. This occurs for inflation forecasts where the combination of the noninformative prior VAR with the subspace shrinkage prior forecasts well. But this result holds only for the one‐quarter‐ahead horizon. The iterated 1‐year‐ahead forecasts are very poor (see Table 3).

Another pattern worth noting is that substantial changes tend to occur during either the financial crisis (around 2009) or the pandemic (2020). The tendency at these times is for the subVAR‐Flat models to do better. Results for GDP growth from larger VARs are particularly striking. The forecast performance of these models was extremely poor up until the pandemic when the subVAR‐Flat models almost caught up to the subVAR‐Minn models. The stronger prior information in the latter is a great benefit in normal times, but in the pandemic, this makes it less able to adjust to the extreme observations which arise. This is because the corresponding predictive density is narrow which helps in tranquil periods while in turbulent times (such as during the pandemic), the variance is too low, rendering outliers less likely under the posterior predictive distribution.

An interesting pattern emerges in the relationship between the priors on ω and the VAR coefficients in VARs of different dimensions. In the small and medium VARs, the two approaches with the same prior for ω (e.g., Minn1 and Flat1) tend to give similar results, depending little on the choice of VAR prior. However, in the larger VARs, it is the VAR prior which matters more. For instance, the lines for Minn0 and Minn1 tend to move together (although inflation forecasts from the XL model are an exception to this pattern) as do Flat0 and Flat1. This is unsurprising as the VAR prior can be expected to be of great importance in larger VARs.

**FIGURE 4:**
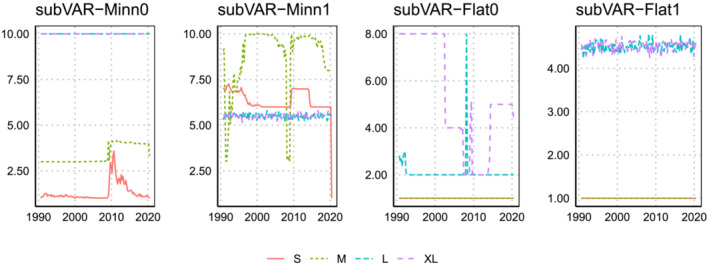
Evolution of the posterior mean of q over the hold‐out period when the marginal likelihood is used to select q, ϑ, and ω.

**FIGURE 5:**
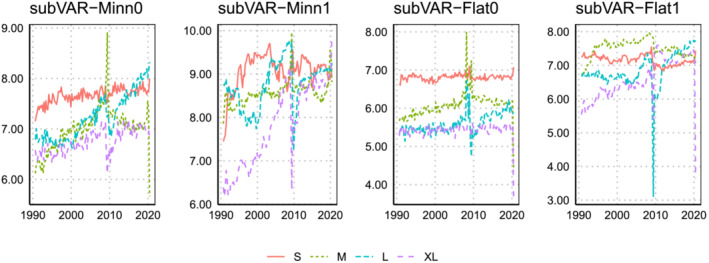
Evolution of the posterior mean of q over the hold‐out period when the BIC over the three focus variables is used to select q, ϑ, and ω.

Figures 4 and 5 plot the posterior mean of q over time for the marginal likelihood‐based and BIC‐based methods, respectively. The figures illustrate some considerable differences between these two methods. In particular, use of the BIC allows for more time variation in the parameter estimates for the XL model suggesting it allows for quicker adjustment to new information. Consider the best‐performing subVAR‐Minn prior with informative prior on ω. For this case, using the marginal likelihood leads to a choice of roughly 5 factors for all time periods for the XL model. But using the BIC, there is more variation over time. For the XL model in particular, the number of factors increases gradually from 6 to 9 before quickly collapsing down to a posterior mean near 6 when the financial crisis hits and subsequently rising up to 9 again.

This pattern does not recur for the lower dimensional models where the marginal likelihood‐based estimates of q tend to be lower. For instance, in the smallest model, the subVAR‐Flat model estimates q=1 for all periods, which contrasts with much larger BIC‐based estimates. These differences most likely arise from the fact that the BIC takes only the three focus variables into account whereas the marginal likelihood searches for an optimal value of q for all elements in $y_{t}$ simultaneously.

**FIGURE 6:**
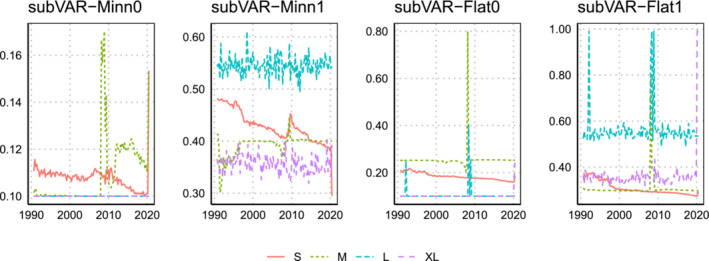
Evolution of the posterior mean of ω over the hold‐out period when the marginal likelihood is used to select q, ϑ, and ω.

**FIGURE 7:**
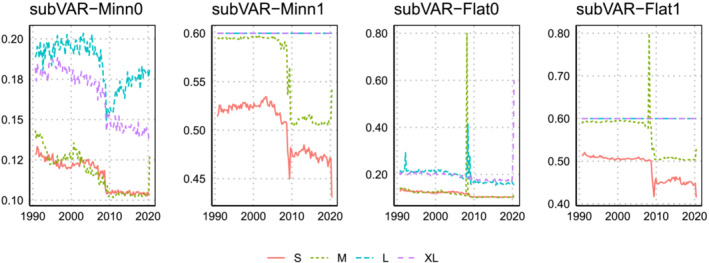
Evolution of the posterior mean of ω over the hold‐out period when the BIC over the three focus variables is used to select q, ϑ, and ω.

Figures 6 and 7 present evidence on the estimation of ω. For the XL and L models, we are finding striking differences between the BIC‐ and marginal likelihood‐based estimates. Note that for subVAR‐Minn model with the informative prior on ω we are finding the posterior mean of ω to be approximately 0.6 when estimated using BIC whereas the marginal likelihood‐based estimates are much lower at approximately 0.25/0.35 for the XL/L models. Hence, the former model is shrinking much more closely to the factor model than the latter.

We can also see the role that the prior for ω has in that estimates using the noninformative prior for ω tend to be substantially lower than those produced using the informative prior. In fact, with rare exceptions, using the noninformative prior never leads to estimates of ω above 0.2. At least in this dataset, it is necessary to use an informative prior for ω to achieve substantial shrinkage towards the factor model. It is interesting to note that, when we do so, we are consistently finding ω to be in the region [0.25,0.60] being far from the region where one would feel confident selecting either the Minnesota prior VAR ( ω=0) or the factor model ( ω=1), thus indicating again the potential benefits of our approach which combines the two.

**FIGURE 8:**
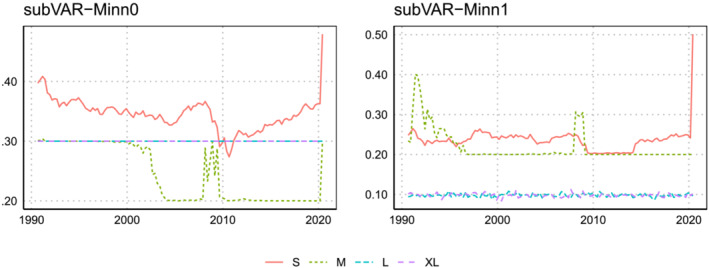
Evolution of the posterior mean of ϑ over the hold‐out period when the marginal likelihood is used to select q, ϑ, and ω.

**FIGURE 9:**
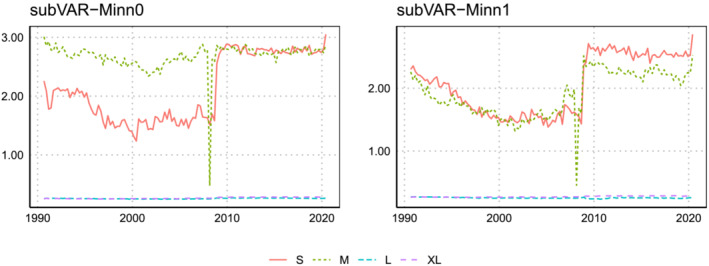
Evolution of the posterior mean of ϑ over the hold‐out period when the BIC over the three focus variables is used to select q, ϑ, and ω.

Finally, we turn to main shrinkage parameter of the Minnesota prior, ϑ. This hyperparameter only appears in the approaches involving the Minnesota prior. Note that smaller values of ϑ imply stronger shrinkage. Posterior means are plotted in Figures 8 and 9.

The most striking pattern here is that using the marginal likelihood leads to much lower estimates of this hyperparameter than using the BIC, especially for the small and medium models. In general, and consistent with Giannone et al. (2015), we find that larger models generally feature smaller values of ϑ (and thus more shrinkage). If we combine this with the fact that the marginal likelihood‐based estimates of ω are lower for these models we have the interesting finding that it is choosing to put more weight on a Minnesota prior VAR with more shrinkage. In contrast, the BIC‐based weights are closer to be a combination of a factor model with a Minnesota prior that is implemented rather loosely.

Another interesting finding is that ϑ tends to sharply increase during the pandemic. This is especially pronounced for small‐ and medium‐sized models. Because the variance of the predictive distribution is positively related to ϑ, larger values of ϑ are (all else being equal) accompanied by wider predictive intervals. This explains why some of the models improve appreciable against the benchmark in 2020.

## 5 EXTENSIONS

### 5.1 Discussion

The methods developed in this paper combine two simple models: the conjugate version of the Minnesota prior VAR with a single shrinkage hyperparameter and a factor model which replaces the factors with PCs. Both of these models are homoskedastic. We did this to draw out all the theoretical insights in a clear and simple way and because, in many empirical contexts, simple approaches such as these have been found to work well (see, e.g., Banbura et al., 2010; Carriero et al., 2015). Furthermore, computation is vastly simplified because analytical results are available and we can avoid the use of MCMC methods. As discussed previously, our PC‐based factor model could be replaced by one which treats factors as unknown latent states in a state space model. Such a model could allow for more sophisticated dynamics for the factors or could include stochastic volatility. The necessary MCMC algorithm is theoretically straightforward to derive, but its computational cost would be substantial in larger models. Accordingly, in this section, we focus on the VAR part of our approach and discuss various extensions which relax some of the assumptions we have made relating to it.

Many recent Bayesian VAR papers have used richer econometric structures. These can be classified in two main categories: other priors and other forms for the error covariance matrix. Here, we discuss using the subVAR prior in the context of such extensions and do additional empirical work for one of the most promising: the asymmetric conjugate prior of Chan (2022).

Global‐local shrinkage priors (e.g., the Horseshoe and Lasso priors) are enjoying an increasing popularity with regressions and VARs. Many of these are conditionally Gaussian (i.e., conditional on some new parameters in the prior they are Gaussian). Estimation proceeds by adding blocks to the MCMC algorithm for drawing these new parameters. Because our Minnesota prior is Gaussian, it is trivial to replace it with any conditionally Gaussian prior. The theory developed in this paper would hold, conditional on the new parameters. Estimation would proceed through an MCMC algorithm which drew these new parameters and then conditional on each draw exploited the subVAR methods developed in this paper.

In a similar fashion, the assumption of homoskedasticity could be relaxed to allow for stochastic volatility. This would lead to an MCMC algorithm which involved drawing the volatilities, and conditional on each draw, the results for the subspace VAR prior developed in this paper could be used. Several forms for stochastic volatility in VARs have been proposed in the literature; see, for instance, Carriero et al. (2019) for a particularly popular form, and the general strategy outlined here would work with any of them.

In sum, many extensions of the conjugate subVAR approach developed in this paper are possible. However, they would require the use of MCMC methods. Provided the likelihood and prior remain Gaussian conditional on some new parameters, the theory derived in Section 2 would hold, conditional on these new parameters.

There are also some specifications of either likelihood or prior that maintain some of the aspects of conjugacy and lead to models which require little or no use of MCMC. The error covariance structure proposed in Chan (2020) is one such example which involves a more flexible likelihood function. It assumes the VAR error covariance matrix is Σ⊗Ω where Ω can be any positive definite matrix. This nests many possible specifications, including a common stochastic volatility model, moving average errors and nonGaussian errors. This Kronecker structure in the likelihood matches up with the Kronecker structure in the conjugate prior leading to derivations which are similar to those in Section 2 of this paper. Roughly speaking, whereas the derivations in Section 2.2 show how the subspace prior leads to a posterior mean which is a combination of the OLS estimate of the VAR with a PC regression, using the model of Chan (2020) leads to a combination of a GLS estimate with a PC regression. Chan (2020) develops a computationally efficient MCMC algorithm for models with this error covariance structure.

### 5.2 Asymmetric conjugate subspace shrinkage

One restrictive feature of our prior is that it involves single parameters governing the number of factors q, the weight on the factor regression ω and shrinkage hyperparameter ϑ which apply to all the equations in the VAR. Allowing for each equation to have its own set of hyperparameters could be a useful extension of our approach. This extension is developed for the VAR in the asymmetric conjugate prior of Chan (2022). A key property of this prior is that it maintains conjugacy and, thus, can avoid the use of MCMC methods. Given the potential for this extension to be empirically useful and its computational practicability, we investigate this extension in more detail in this subsection.

Complete details of the asymmetric conjugate prior are available in Chan (2022), and we follow them precisely. However, the main aspects can be described succinctly. The reduced form VAR in (1) can be transformed into a structural VAR by multiplying both sides of it by $A_{0}$ which is obtained from the decomposition $\Sigma =A_{0}^{-1}D{\left(A_{0}^{-1}\right)}^{\prime }$ where $A_{0}$ is lower triangular with ones on the diagonal and D is a diagonal matrix. Because the errors in the structural VAR are independent of one another, Bayesian estimation can proceed one equation at a time. The asymmetric conjugate prior is simply a set of individual conjugate priors for each of the M equations in the VAR. Because each of these priors has its own shrinkage parameter, the assumption of a single shrinkage parameter can be relaxed.

The asymmetric conjugate prior VAR with subspace shrinkage combines the conjugate prior for a regression model with a PC regression in the same manner as for our subspace VAR but does so one equation at a time. All the formulae in Section 2 still hold (with the obvious redefinitions of data matrices and parameters) but for each equation individually. Our prior hyperparameters will now vary across equations and be $\omega_{i}$ and $\vartheta_{i}$ and $q_{i}$ for i=1…M. The priors on these hyperparameters are the same as in the symmetric conjugate VAR with the main difference that our prior on $\omega_{i}$ is always set to be uninformative.

Assuming that the hyperparameters differ across equations substantially increases the computational burden and memory requirements. This is because the Monte Carlo integration strategy described in Section 2.4 has to be done in 3×M dimensions instead of three dimensions. Because precomputing and saving all posterior quantities in the same way we did for the symmetric model is extremely memory intensive, we rely on an plug‐in approach by first computing the marginal likelihood for each $\omega_{i}$, $q_{i}$, and $\vartheta_{i}$ and then selecting the one that maximizes the marginal likelihood per equation. But other than this, no posterior simulation is required, and thus, we can even handle the XL dataset with the asymmetric conjugate prior VAR with subspace shrinkage, which we denote subVAR‐Asym.

Table 4 repeats the forecasting exercise of Section 4.3, comparing the forecast performance of the subVAR‐Asym to the benchmark Minnesota prior VAR and our previously best‐performing subVAR approach: subVAR‐Minn1. Overall, this new prior, based on the asymmetric conjugate prior, is not appreciably better than the one based on the Minnesota prior and, in fact, in most cases is slightly worse. But it is competitive and in some cases (e.g., density forecasts of inflation) does outperform subVAR‐Minn1.

**TABLE 4:** Forecasting results: Comparison of subVAR‐Asym to other approaches.

		FEDFUNDS				CPIAUCSL			GDPC1	
		subVAR‐Minn1	subVAR‐Asym	BVAR	subVAR‐Minn1	subVAR‐Asym	BVAR	subVAR‐Minn0	subVAR‐Asym	BVAR
One‐quarter‐ahead										
	S	0.93	1.02	0.58	0.98	0.98	1.22	0.98	1.00	1.14
		( 0.03)	(−0.15)	(−0.87)	( 0.11)	(−0.02)	(−2.01)	(−0.03)	( 0.05)	(−1.62)
	M	0.91	1.01	0.65	0.97	0.95	1.26	0.99	0.99	1.14
		( 0.07)	(−0.26)	(−0.86)	( 0.12)	( 0.58)	(−2.33)	( 0.01)	( 0.15)	(−1.65)
	L	0.62	0.82	0.74	0.86	0.93	1.32	0.93	0.94	1.20
		( 0.51)	(−0.21)	(−1.24)	( 2.27)	( 2.51)	(−4.09)	( 0.11)	( 0.07)	(−1.73)
	XL	0.68	0.88	0.74	0.87	0.94	1.35	0.92	0.94	1.21
		( 1.53)	( 0.81)	(−2.27)	( 2.66)	( 3.56)	(−5.20)	( 0.53)	( 0.47)	(−2.27)
One‐year‐ahead										
	S	1.03	1.05	0.48	1.00	1.00	1.14	1.00	1.00	1.12
		( 0.07)	(−0.30)	(−1.07)	(−0.01)	(−0.08)	(−1.60)	( 0.01)	(−0.04)	(−1.63)
	M	0.96	1.03	0.53	1.00	0.99	1.15	0.99	1.01	1.12
		( 0.08)	(−0.49)	(−1.04)	(−0.12)	( 0.15)	(−1.75)	(−0.02)	(−0.05)	(−1.60)
	L	0.66	0.91	0.69	0.95	0.97	1.18	0.98	0.97	1.13
		( 0.32)	(−0.69)	(−1.56)	( 0.05)	(−0.41)	(−1.57)	(−0.06)	(−0.24)	(−1.66)
	XL	0.54	0.82	0.86	0.95	0.95	1.19	0.98	0.99	1.13
		(−0.88)	(−1.46)	(−1.45)	(−0.72)	(−1.10)	(−1.54)	(−1.06)	(−0.85)	(−1.51)

*Note*: subVAR‐Minn1 denotes the VAR coupled with a combination between subspace and Minnesota shrinkage and an informative prior on ω. subVAR‐Asym denotes the asymmetric conjugate prior VAR with a combination between subspace and Minnesota shrinkage and an uninformative prior on ω. The numbers are relative root mean squared forecast errors (RMSFEs) between a given model and the Minnesota VAR while the numbers in parentheses are differences in average log predictive likelihoods (LPLs) to the Minnesota VAR. The numbers in the BVAR columns include the actual RMSFEs and LPLs of the Minnesota VAR.

**FIGURE 10:**
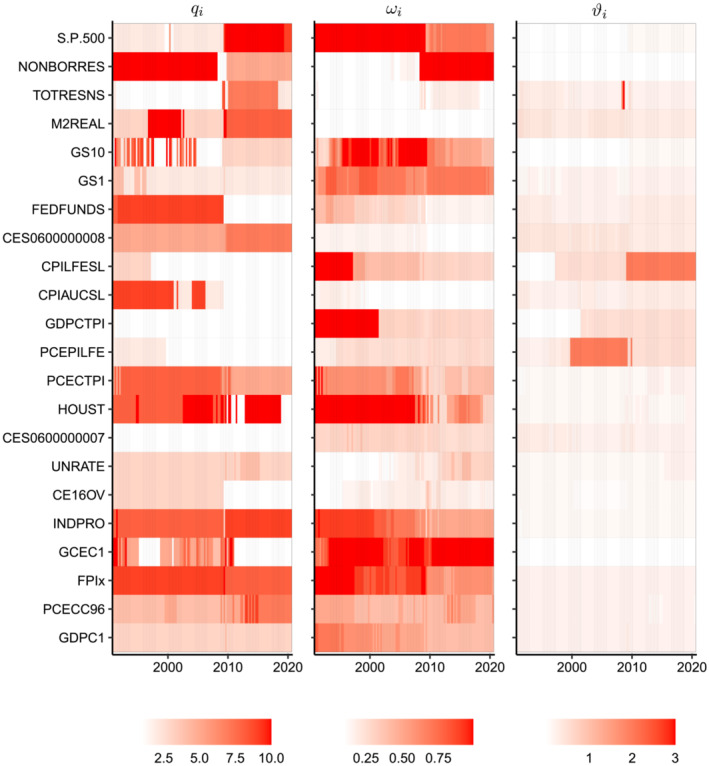
Evolution of equation‐specific hyperparameters $q_{i}$, $\omega_{i}$, and q for the medium‐sized VAR over the hold‐out period.

To dig a little deeper into the performance of subVAR‐Asym, Figure 10 presents a heatmap of the posterior means of the prior hyperparameters from the recursive forecasting exercise for the medium‐sized model. The key point worth noting is that the hyperparameter estimates do vary substantially across the equations in the VAR. This holds true particularly for $q_{i}$ and $\omega_{i}$. For instance, results for $\omega_{i}$ imply that most of the equations are estimated using a combination of shrinkage prior and factor approaches, but some equations (e.g., GCEC1) are close to being purely PC regression models and others (e.g., CPIAUCSL) are close to being Bayesian regressions with shrinkage priors. The ability to automatically select a factor model for some equations and a shrinkage prior for others is a potentially useful feature of the model which uses the asymmetric conjugate prior with subspace shrinkage. To our knowledge, it is not available in any other Bayesian multivariate time series model.

There is also appreciable variation in the estimated prior hyperparameters over time in many cases. This shows, for instance, how the forecasting model can switch from being a PC regression to being a regression with shrinkage prior in a data‐based fashion. This model switching ability is another potentially useful feature of this approach that is not possible with other Bayesian VAR priors.

## 6 CONCLUSIONS

Macroeconomic researchers with large datasets have traditionally been forced to make a choice between a large VAR or a factor model. In this paper, we have shown how to combine the two. We have developed a subspace prior for the VAR which shrinks towards a factor model. This prior model assumes that the latent factors are estimated through the PCs of the full‐data matrix X. A parameter, ω, controls the degree of shrinkage, and we have developed methods for estimating it from the data. Thus, we have developed a Bayesian methodology for averaging a large VAR with a factor model or choosing between them.

We illustrate our approach using synthetic and real data. In simulations, we show that our subspace prior accurately detects whether the data arise from a factor model or an unrestricted VAR. In case the DGP is a factor model and the true number of factors is relatively small, our model accurately selects the true number of factors (irrespective of the model size). If the DGP features a large number of factors (and the number of time series is very large), our approach underestimates the true number of factors. In a forecasting exercise involving a large number of macroeconomic variables, we demonstrate the benefits of combining the two model classes using our subspace VAR. Using subspace shrinkage in combination with a Minnesota prior often yields more precise forecasts than the ones obtained from either the factor model or the VAR.

### OPEN RESEARCH BADGES

The dataset and replication files are available in the Journal of Applied Econometrics Replication Archive: DOI: 10.15456/jae.2023031.1448252680.

## Supporting information

This article has been awarded Open Data Badge for making publicly available the digitally‐shareable data necessary to reproduce the reported results. Data is available at Open Science Framework.

Appendix.pdf

## Data Availability

This article has been awarded Open Data Badge for making publicly available the digitally‐shareable data necessary to reproduce the reported results. Data is available at Open Science Framework.

## Footnotes

1 All the factors in this paper are PCs. However, it is worth noting that it would be possible to treat the factors as unknown states and use Bayesian state space methods. The cost of the doing this would be a large increase in the computational burden due to the need to use posterior simulation methods.

2 For brevity, we exclude deterministic terms. In our empirical work, we include an intercept.

3 Note that if we were to set $\Phi_{0}=0_{T\times T}$, then the prior would reduce to a standard g‐prior with hyperparameter ω/(1−ω).

4 We stress that the approximation error being referred to is only in the theoretical relationship given in (7) and not in our posterior. There is no approximation error in the latter.

5 For more details on the DGP, see Section 3.

6 Here, we will discuss how to carry out Bayesian inference on these three prior hyperparameters. Note that our basic subspace shrinkage prior given by Equation (4) is obtained as a special case by letting ϑ→∞ and Bayesian inference on the two remaining prior hyperparameters can be carried out as a special case of methods for the subVAR‐Minn prior.

7 If the prior hyperparameters were fixed, as opposed to being treated as unknown parameters, this would be the marginal likelihood.

8 The Ledermann bound is defined as the smallest solution $L^{*}$ to the equation ${\left(K-L^{*}\right)}^{2}\geq K+L^{*}$.

9 We stress that this DFM is used only as a DGP. The factors in our subspace VAR priors are always based on PCs.
