# Supplementary material for: Subspace shrinkage in conjugate Bayesian vector autoregressions
Source: J Appl Econ (Chichester Engl). 2023 Mar 15;38(4):556–76. doi: 10.1002/jae.2966 (PMC10947394; doi:10.1002/jae.2966)
Supplement: Supplementary file 1 — Appendix.pdf [file JAE-38-556-s001.pdf]

A Technical appendix

A.1 Properties of the subspace shrinkage Prior

To see how ω shrinks the VAR towards the factor model, it is convenient to exploit the fact that if the rank of $\boldsymbol{X}$ is K, the matrix $\boldsymbol{X}$ and the matrix $\boldsymbol{F}_K$ (i.e., the first K PCs of $\boldsymbol{X}$) span the same column space C. Moreover, notice that $\boldsymbol{F}_K = (\boldsymbol{F}_q, \boldsymbol{F}_{(q+1):K})$ with $\boldsymbol{F}_{(q+1):K}$ storing the final $K-q$ principal components of $\boldsymbol{X}$. Using these definitions and the result that $C(\boldsymbol{X}) = C(\boldsymbol{F}_K)$, the corresponding projection matrices coincide:

$$\boldsymbol{X}\left(\boldsymbol{X}'\boldsymbol{X} + \frac{\omega}{1-\omega}\boldsymbol{X}'(\boldsymbol{I}_T - \boldsymbol{\Phi}_0)\boldsymbol{X}\right)^{-1}\boldsymbol{X}' \tag{10}$$

$$= \boldsymbol{F}_K\left(\boldsymbol{F}_K'\boldsymbol{F}_K + \frac{\omega}{1-\omega}\boldsymbol{F}_K'(\boldsymbol{I}_T - \boldsymbol{\Phi}_0)\boldsymbol{F}_K\right)^{-1}\boldsymbol{F}_K'. \tag{11}$$

Notice that conditional on a standard normalization, we have that $\boldsymbol{F}_K'\boldsymbol{F}_K = \boldsymbol{I}_K$ and $\boldsymbol{F}_q'\boldsymbol{F}_{(q+1):K} = \boldsymbol{0}$. This allows us to rewrite Eq. (11) as:

$$\boldsymbol{F}_K\left(\boldsymbol{I}_K + \frac{\omega}{1-\omega}\left[\boldsymbol{I}_K - \begin{pmatrix}\boldsymbol{I}_q & \boldsymbol{0}\\ \boldsymbol{0} & \boldsymbol{0}\end{pmatrix}\right]\right)^{-1}\boldsymbol{F}_K' \tag{12}$$

$$= (\boldsymbol{F}_q,\ \boldsymbol{F}_{q+1:K})\begin{pmatrix}\boldsymbol{I}_q & \boldsymbol{0}\\ \boldsymbol{0} & (1-\omega)\boldsymbol{I}_{K-q}\end{pmatrix}\begin{pmatrix}\boldsymbol{F}_q'\\ \boldsymbol{F}_{q+1:K}'\end{pmatrix} \tag{13}$$

$$= \boldsymbol{\Phi}_0 + (1-\omega)\boldsymbol{\Phi}_1, \tag{14}$$

with $\boldsymbol{\Phi}_1 = \boldsymbol{F}_{q+1:K}(\boldsymbol{F}_{q+1:K}'\boldsymbol{F}_{q+1:K})^{-1}\boldsymbol{F}_{q+1:K}'$. It is straightforward to show that

$$\boldsymbol{\Phi} = \boldsymbol{\Phi}_0 + \boldsymbol{\Phi}_1,$$

where $\boldsymbol{\Phi} = \boldsymbol{X}(\boldsymbol{X}'\boldsymbol{X})^{-1}\boldsymbol{X}'$ is the projection matrix of $\boldsymbol{X}$. Thus, we can substitute $\boldsymbol{\Phi}_1 = \boldsymbol{\Phi} - \boldsymbol{\Phi}_0$ in Eq. (14) and multiply from the right with $\boldsymbol{Y}$ to arrive at:

$$\mathbb{E}(\boldsymbol{X}\boldsymbol{A}|\boldsymbol{Y},\omega) = \omega\boldsymbol{\Phi}_0\boldsymbol{Y} + (1-\omega)\boldsymbol{\Phi}\boldsymbol{Y}, \tag{15}$$

which shows that the posterior mean of the regression function is a convex combination of the VAR fit, $\boldsymbol{\Phi}\boldsymbol{Y}$, and the fit of the PC regression, $\boldsymbol{\Phi}_0\boldsymbol{Y}$.

A.2 Our implementation of the Minnesota prior

In our empirical work we set the dummies as follows (see Banbura, Giannone, and Reichlin, 2010):

$$\underline{\boldsymbol{Y}} = \begin{pmatrix} \text{diag}(\underline{a}_1\hat{\sigma}_1, \ldots, \underline{a}_M\hat{\sigma}_M)/\vartheta \\ \mathbf{0}_{M(p-1)\times M} \\ \text{diag}(\hat{\sigma}_1, \ldots, \hat{\sigma}_M) \\ \mathbf{0}_{1\times M} \end{pmatrix}, \quad \underline{\boldsymbol{X}} = \begin{pmatrix} \boldsymbol{J}_p \otimes \text{diag}(\hat{\sigma}_1, \ldots, \hat{\sigma}_M)/\vartheta & \mathbf{0}_{K\times 1} \\ \mathbf{0}_{M\times K} & \mathbf{0}_{M\times 1} \\ \mathbf{0}_{1\times K} & \kappa \end{pmatrix},$$

with $\hat{\sigma_j}$ $(j = 1, \ldots, M)$ denoting the OLS residual standard deviation of an AR(p) model for y_{jt}, the jth variable in $\mathbf{Y}_t$, $\underline{a}_j$ is the jth diagonal element of $\underline{\boldsymbol{A}}$, and $\boldsymbol{J}_p = \text{diag}(1, \ldots, p)$. Notice that this set of dummies includes the prior for the intercept which depends on the hyperparameter κ. κ is set to a very small number (in our empirical work it equals 0.001), leading to a weakly informative prior for the intercepts. Notice that the prior precision of the Minnesota prior is given by $\underline{\boldsymbol{X}}'\underline{\boldsymbol{X}}$ and the prior mean is equal to the OLS estimate based on the dummy observations: $\underline{\boldsymbol{A}} = (\underline{\boldsymbol{X}}'\underline{\boldsymbol{X}})^{-1}\underline{\boldsymbol{X}}'\underline{\boldsymbol{Y}}$.

B Additional simulation results

In Sub-section 3 we showed that our approach is able to recover the true number of factors as long as M is not too large. We stated that one of the reasons why we under-estimate the true number of factors in large dimensional models is due to the fact that the VAR is capable of mimicking factor dynamics in finite samples and the likelihood is not strongly informative about whether a factor model or a VAR should be used to model $\boldsymbol{y}_t$. If the number of observations, however, increases, our approach should be able to infer the true number of factors. We provide simulation evidence to support this claim in Table 5. This table is the same as Table 1 with two main differences. First, we drop the models which have an informative prior on ω for brevity reasons. Second, and more importantly, we increase the length of the time series and set $T = 2500$.

Table 5: Simulation results for differing values of q and M and $T = 2500$. Averages across 100 replications from the factor model DGP

	subVAR-Minn0				subVAR-Flat0			
$q =$	1	3	6	8	1	3	6	8
Posterior mean of q								
$M = 30$	1.00	3.00	6.32	8.00	1.00	3.04	6.16	8.00
$M = 60$	1.00	3.00	6.00	8.00	1.00	3.00	6.00	8.00
$M = 120$	1.00	3.00	6.00	8.00	1.00	3.00	6.00	8.00
Posterior mean of ω								
$M = 30$	0.99	0.99	0.99	0.82	0.99	0.99	0.99	0.81
$M = 60$	0.99	0.99	0.99	0.99	0.99	0.99	0.99	0.99
$M = 120$	0.99	0.99	0.99	0.99	0.99	0.99	0.99	0.99

Notes: subVAR denotes the VAR coupled with the subspace shrinkage prior, Minn is the combination between subspace and Minnesota shrinkage while flat is the subspace shrinkage prior without additional shrinkage. Both models feature a flat prior on ω. Each number is based on computing the mean of posterior medians across 100 replications from the respective DGPs. For q, we use the posterior median as our point estimate while for ω we use the posterior mean.

The table suggests that if the number of observations becomes large, our model detects the true number of factors very accurately. For all model sizes, the estimated number of factors is never far away from the true number of factors. This finding suggests that the accuracy of our approach, as expected, strongly depends on the relationship between the number of coefficients and the length of the time series. Since the dimension of the state space grows quadratically in M, substantial information in the likelihood is required to infer the true number of factors for $M = 120$.

C Data Appendix

Table 6: Description of the Dataset

ID	FRED Code	Description	Transformation Codes	S	M	L	XL
1	GDPC1	Real Gross Domestic Product	5	X	X	X	X
2	PCECC96	Real Personal Consumption Expenditures	5		X	X	X
3	PCDGx	Real personal consumption expenditures: Durable goods	5				X
4	PCESVx	Real Personal Consumption Expenditures: Services	5			X	X
5	PCNDx	Real Personal Consumption Expenditures: Nondurable Goods	5			X	X
6	GPDIC1	Real Gross Private Domestic Investment	5			X	X
7	FPIx	Real private fixed investment	5		X	X	X
8	Y033RC1Q027SBEAx	Real Gross Private Domestic Investment: Fixed Investment: Nonresidential Equipment	5			X	X
9	PNFIx	Real private fixed investment: Nonresidential	5			X	X
10	PRFIx	Real private fixed investment: Residential	5			X	X
11	A014RE1Q156NBEA	Shares of gross domestic product: Gross private domestic investment: Change in private inventories	1			X	X
12	GCEC1	Real Government Consumption Expenditures and Gross Investment	5		X	X	X
13	A823RL1Q225SBEA	Real Government Consumption Expenditures and Gross Investment: Federal	1				X
14	FGRECPTx	Real Federal Government Current Receipts	5				X
15	SLCEx	Real government state and local consumption expenditures	5				X
16	EXPGSC1	Real Exports of Goods and Services	5			X	X
17	IMPGSC1	Real Imports of Goods and Services	5			X	X
18	DPIC96	Real Disposable Personal Income	5			X	X
19	OUTNFB	Nonfarm Business Sector: Real Output	5				X
20	OUTBS	Business Sector: Real Output	5				X
21	INDPRO	IP:Total index Industrial Production Index (Index 2012=100)	5		X	X	X
22	IPFINAL	IP:Final products Industrial Production: Final Products (Market Group) (Index 2012=100)	5			X	X
23	IPCONGD	IP:Consumer goods Industrial Production: Consumer Goods (Index 2012=100)	5			X	X
24	IPMAT	Materials (Index 2012=100)	5				X
25	IPDMAT	Durable Materials (Index 2012=100)	5				X
26	IPNMAT	Nondurable Materials (Index 2012=100)	5				X
27	IPDCONGD	Durable Consumer Goods (Index 2012=100)	5				X
28	IPB51110SQ	Durable Goods: Automotive products (Index 2012=100)	5				X
29	IPNCONGD	Nondurable Consumer Goods (Index 2012=100)	5				X
30	IPBUSEQ	Business Equipment (Index 2012=100)	5				X
31	IPB51220SQ	Consumer energy products (Index 2012=100)	5				X
32	CUMFNS	Capacity Utilization: Manufacturing (SIC) (Percent of Capacity)	1				X
33	IPMANSICS	Industrial Production: Manufacturing (SIC) (Index 2012=100)	5				X
34	IPB51222S	Industrial Production: Residential Utilities (Index 2012=100)	5				X
35	IPFUELS	Industrial Production: Fuels (Index 2012=100)	5				X
36	PAYEMS	Emp:Nonfarm All Employees: Total nonfarm (Thousands of Persons)	5			X	X
37	USPRIV	All Employees: Total Private Industries (Thousands of Persons)	5				X
38	MANEMP	All Employees: Manufacturing (Thousands of Persons)	5				X
39	SRVPRD	All Employees: Service-Providing Industries (Thousands of Persons)	5				X
40	USGOOD	All Employees: Goods-Producing Industries (Thousands of Persons)	5				X
41	DMANEMP	All Employees: Durable goods (Thousands of Persons)	5				X
42	NDMANEMP	All Employees: Nondurable goods (Thousands of Persons)	5				X
43	USCONS	All Employees: Construction (Thousands of Persons)	5				X
44	USEHS	All Employees: Education & Health Services (Thousands of Persons)	5				X
45	USFIRE	All Employees: Financial Activities (Thousands of Persons)	5				X
46	USINFO	All Employees: Information Services (Thousands of Persons)	5				X
47	USPBS	All Employees: Professional & Business Services (Thousands of Persons)	5				X
48	USLAH	All Employees: Leisure & Hospitality (Thousands of Persons)	5				X
49	USSERV	All Employees: Other Services (Thousands of Persons)	5				X
50	USMINE	All Employees: Mining and logging (Thousands of Persons)	5				X
51	USTPU	All Employees: Trade, Transportation & Utilities (Thousands of Persons)	5				X
52	USGOVT	All Employees: Government (Thousands of Persons)	5				X
53	USTRADE	All Employees: Retail Trade (Thousands of Persons)	5				X
54	USWTRADE	All Employees: Wholesale Trade (Thousands of Persons)	5				X
55	CES9091000001	All Employees: Government: Federal (Thousands of Persons)	5				X
56	CES9092000001	All Employees: Government: State Government (Thousands of Persons)	5				X
57	CES9093000001	All Employees: Government: Local Government (Thousands of Persons)	5				X
58	CE16OV	Civilian Employment (Thousands of Persons)	5	X	X	X	X
59	CIVPART	Civilian Labor Force Participation Rate (Percent)	2				X
60	UNRATE	Civilian Unemployment Rate (Percent)	2	X	X	X	X
61	UNRATESTx	Unemployment Rate less than 27 weeks (Percent)	2				X
62	UNRATELTx	Unemployment Rate for more than 27 weeks (Percent)	2			X	X
63	LNS14000012	Unemployment Rate - 16 to 19 years (Percent)	2				X
64	LNS14000025	Unemployment Rate - 20 years and over, Men (Percent)	2				X
65	LNS14000026	Unemployment Rate - 20 years and over, Women (Percent)	2				X
66	UEMPLT5	Number of Civilians Unemployed - Less Than 5 Weeks (Thousands of Persons)	5				X
67	UEMP5TO14	Number of Civilians Unemployed for 5 to 14 Weeks (Thousands of Persons)	5				X
68	UEMP15T26	Number of Civilians Unemployed for 15 to 26 Weeks (Thousands of Persons)	5				X
69	UEMP27OV	Number of Civilians Unemployed for 27 Weeks and Over (Thousands of Persons)	5				X
70	AWHMAN	Average Weekly Hours of Production and Nonsupervisory Employees: Manufacturing (Hours)	1			X	X
71	AWOTMAN	Average Weekly Overtime Hours of Production and Nonsupervisory Employees: Manufacturing (Hours)	2			X	X
72	HWIx	Help-Wanted Index	1				X
73	CES0600000007	Average Weekly Hours of Production and Nonsupervisory Employees: Goods-Producing	2	X	X	X	X
74	CLAIMSx	Initial Claims	5			X	X
75	HOUST	Housing Starts: Total: New Privately Owned Housing Units Started	5		X	X	X
76	HOUST5F	Privately Owned Housing Starts: 5-Unit Structures or More	5				X
77	HOUSTMW	Housing Starts in Midwest Census Region (Thousands of Units)	5				X
78	HOUSTNE	Housing Starts in Northeast Census Region (Thousands of Units)	5				X
79	HOUSTS	Housing Starts in South Census Region (Thousands of Units)	5				X
80	HOUSTW	Housing Starts in West Census Region (Thousands of Units)	5				X
81	RSAFSx	Real Retail and Food Services Sales (Millions of Chained 2012 Dollars)	5			X	X
82	AMDMNOx	Real Manufacturers New Orders: Durable Goods (Millions of 2012 Dollars)	5				X
83	AMDMUOx	Real Value of Manufacturers Unfilled Orders for Durable Goods Industries	5				X
84	BUSINVx	Total Business Inventories (Millions of Dollars)	5				X
85	ISRATIOx	Total Business: Inventories to Sales Ratio	2				X
86	PCECTPI	Personal Consumption Expenditures: Chain-type Price Index	6	X	X	X	X
87	PCEPILFE	Personal Consumption Expenditures Excluding Food and Energy	6	X	X	X	X
88	GDPCTPI	Gross Domestic Product: Chain-type Price Index	6	X	X	X	X
89	GPDICTPI	Gross Private Domestic Investment: Chain-type Price Index	6			X	X
90	IPDBS	Business Sector: Implicit Price Deflator (Index 2012=100)	6			X	X

ID	FRED Code	Description	Transformation Codes	S	M	L	XL
91	DGDSRG3Q086SBEA	Personal consumption expenditures: Goods	6			X	X
92	DDURRG3Q086SBEA	Personal consumption expenditures: Durable goods	6			X	X
93	DSERRG3Q086SBEA	Personal consumption expenditures: Services	6			X	X
94	DNDGRG3Q086SBEA	Personal consumption expenditures: Nondurable goods	6			X	X
95	DHCERG3Q086SBEA	Personal consumption expenditures: Services: Household consumption expenditures	6				X
96	DMOTRG3Q086SBEA	Personal consumption expenditures: Durable goods: Motor vehicles and parts	6				X
97	DFDHRG3Q086SBEA	Personal consumption expenditures: Durable goods: Furnishings and durable household equipment	6				X
98	DREQRG3Q086SBEA	Personal consumption expenditures: Durable goods: Recreational goods and vehicles	6				X
99	DODGRG3Q086SBEA	Personal consumption expenditures: Durable goods: Other durable goods	6				X
100	DFXARG3Q086SBEA	Personal consumption expenditures: Nondurable goods: Food and beverages purchased for off-premises consumption	6				X
101	DCLORG3Q086SBEA	Personal consumption expenditures: Nondurable goods: Clothing and footwear	6				X
102	DGOERG3Q086SBEA	Personal consumption expenditures: Nondurable goods: Gasoline and other energy goods	6				X
103	DONGRG3Q086SBEA	Personal consumption expenditures: Nondurable goods: Other nondurable goods	6				X
104	DHUTRG3Q086SBEA	Personal consumption expenditures: Services: Housing and utilities	6				X
105	DHLCRG3Q086SBEA	Personal consumption expenditures: Services: Health care	6				X
106	DTRSRG3Q086SBEA	Personal consumption expenditures: Transportation services	6				X
107	DRCARG3Q086SBEA	Personal consumption expenditures: Recreation services	6				X
108	DFSARG3Q086SBEA	Personal consumption expenditures: Services: Food services and accomodations	6				X
109	DIFSRG3Q086SBEA	Personal consumption expenditures: Financial services and insurance	6				X
110	DOTSRG3Q086SBEA	Personal consumption expenditures: Other services	6				X
111	CPIAUCSL	Consumer Price Index for All Urban Consumers: All Items	6	X	X	X	X
112	CPILFESL	Consumer Price Index for All Urban Consumers: All Items Less Food & Energy	6	X	X	X	X
113	WPSFD49207	Producer Price Index by Commodity for Finished Goods	6			X	X
114	PPIACO	Producer Price Index for All Commodities	6			X	X
115	WPSFD49502	Producer Price Index by Commodity for Finished Consumer Goods	6				X
116	WPSFD4111	Producer Price Index by Commodity for Finished Consumer Foods	6				X
117	PPIIDC	Producer Price Index by Commodity Industrial Commodities	6				X
118	WPSID61	Producer Price Index by Commodity Intermediate Materials: Supplies & Components	6				X
119	WPU0561	Producer Price Index by Commodity for Fuels and Related Products and Power	5			X	X
120	OILPRICEx	Real Crude Oil Prices: West Texas Intermediate (WTI) - Cushing, Oklahoma	5			X	X
121	WPSID62	Producer Price Index: Crude Materials for Further Processing	6				X
122	PPICMM	Producer Price Index: Commodities: Metals and metal products: Primary nonferrous metals	6				X
123	CPIAPPSL	Consumer Price Index for All Urban Consumers: Apparel	6			X	X
124	CPITRNSL	Consumer Price Index for All Urban Consumers: Transportation	6			X	X
125	CPIMEDSL	Consumer Price Index for All Urban Consumers: Medical Care	6			X	X
126	CUSR0000SAC	Consumer Price Index for All Urban Consumers: Commodities	6			X	X
127	CES2000000008x	Real Average Hourly Earnings of Production and Nonsupervisory Employees: Construction	5			X	X
128	CES3000000008x	Real Average Hourly Earnings of Production and Nonsupervisory Employees: Manufacturing	5			X	X
129	COMPRNFB	Nonfarm Business Sector: Real Compensation Per Hour (Index 2012=100)	5			X	X
130	CES0600000008	Average Hourly Earnings of Production and Nonsupervisory Employees:	6	X	X	X	X
131	FEDFUNDS	Effective Federal Funds Rate (Percent)	2	X	X	X	X
132	TB3MS	3-Month Treasury Bill: Secondary Market Rate (Percent)	2			X	X
133	TB6MS	6-Month Treasury Bill: Secondary Market Rate (Percent)	2			X	X
134	GS1	1-Year Treasury Constant Maturity Rate (Percent)	2		X	X	X
135	GS10	10-Year Treasury Constant Maturity Rate (Percent)	2	X	X	X	X
136	AAA	Moody's Seasoned Aaa Corporate Bond Yield (Percent)	2				X
137	BAA	Moody's Seasoned Baa Corporate Bond Yield (Percent)	2			X	X
138	BAA10YM	Moody's Seasoned Baa Corporate Bond Yield Relative to Yield on 10-Year Treasury	1				X
139	TB6M3Mx	6-Month Treasury Bill Minus 3-Month Treasury Bill, secondary market (Percent)	1			X	X
140	GS1TB3Mx	1-Year Treasury Constant Maturity Minus 3-Month Treasury Bill, secondary market	1			X	X
141	GS10TB3Mx	10-Year Treasury Constant Maturity Minus 3-Month Treasury Bill, secondary market	1			X	X
142	CPF3MTB3Mx	3-Month Commercial Paper Minus 3-Month Treasury Bill, secondary market	1			X	X
143	GS5	5-Year Treasury Constant Maturity Rate	2			X	X
144	TB3SMFFM	3-Month Treasury Constant Maturity Minus Federal Funds Rate	1			X	X
145	T5YFFM	5-Year Treasury Constant Maturity Minus Federal Funds Rate	1			X	X
146	AAAFFM	Moody's Seasoned Aaa Corporate Bond Minus Federal Funds Rate	1			X	X
147	M1REAL	Real M1 Money Stock	5			X	X
148	M2REAL	Real M2 Money Stock	5		X	X	X
149	BUSLOANSx	Real Commercial and Industrial Loans, All Commercial Banks	5			X	X
150	CONSUMERx	Real Consumer Loans at All Commercial Banks	5			X	X
151	NONREVSLx	Total Real Nonrevolving Credit Owned and Securitized, Outstanding	5			X	X
152	REALLNx	Real Real Estate Loans, All Commercial Banks	5			X	X
153	TOTALSLx	Total Consumer Credit Outstanding	5			X	X
154	TOTRESNS	Total Reserves of Depository Institutions	6		X	X	X
155	NONBORRES	Reserves Of Depository Institutions, Nonborrowed	7		X	X	X
156	DTCOLNVHFNM	Consumer Motor Vehicle Loans Outstanding Owned by Finance Companies	6				X
157	DTCTHFNM	Total Consumer Loans and Leases Outstanding Owned and Securitized by Finance Companies	6				X
158	INVEST	Securities in Bank Credit at All Commercial Banks	6				X
159	TABSHNOx	Real Total Assets of Households and Nonprofit Organizations	5				X
160	EXSZUSx	Switzerland / U.S. Foreign Exchange Rate	5			X	X
161	EXJPUSx	Japan / U.S. Foreign Exchange Rate	5			X	X
162	EXUSUKx	U.S. / U.K. Foreign Exchange Rate	5			X	X
163	EXCAUSx	Canada / U.S. Foreign Exchange Rate	5			X	X
164	S.P.500	S&P's Common Stock Price Index: Composite	5		X	X	X
165	S.P..indust	S&P'sCommon Stock Price Index: Industrials	5				X
166	S.P.div.yield	S&P's Composite Common Stock: Dividend Yield	2				X

Notes: This table provides an overview of the dataset employed. The transformation codes are applied to each time series $\boldsymbol{Y}_j$ and described in McCracken and Ng (2020): (1) no transformation; (2) Δy_{jt}; (3) $\Delta^2 y_{jt}$; (4) $\log(y_{jt})$; (5) $\Delta \log(y_{jt})$; (6) $\Delta^2 \log(y_{jt})$; (7) $\Delta(y_{jt}/y_{jt-1} - 1)$. 'X' marks the inclusion of a variable into one of the datasets.
